# Synthesis, Molecular Structure, Spectral Properties and Antifungal Activity of Polymethylene-α,ω-bis(*N,N*- dimethyl-*N*-dodecyloammonium Bromides)

**DOI:** 10.3390/molecules16010319

**Published:** 2011-01-05

**Authors:** Bogumił Brycki, Iwona Kowalczyk, Anna Koziróg

**Affiliations:** 1 Laboratory of Microbiocides Chemistry, Faculty of Chemistry, Adam Mickiewicz University, Grunwaldzka 6, 60-780 Poznań, Poland; 2 Institute of Technology Fermentation and Microbiology, Technical University of Lodz, Wolczanska 171/173, 90-924 Lodz, Poland

**Keywords:** polymethylene-α,ω-bis(*N,N*-dimethyl-*N*-dodecyloammonium bromides), FTIR and NMR spectra, DFT calculations, antifungal activity

## Abstract

Hexamethylene-1,6-bis-(*N,N*-dimethyl-*N*-dodecylammonium bromide) (**1**), pentamethylene-1,5-bis(*N,N*-dimethyl-*N*-dodecylammonium bromide) (**2**), tetramethylene-1,4-bis(*N,N*-dimethyl-*N*-dodecylammonium bromide) (**3**), trimethylene-1,3-bis(*N,N*-dimethyl-*N*-dodecylammonium bromide) (**4**) and ethylene-1,2-bis(*N,N*-dimethyl-*N*-dodecylammonium bromide) (**5**) have been obtained and characterized by FTIR and NMR spectroscopy. DFT calculations have also been carried out. The optimized bond lengths, bond angles and torsion angles calculated by Hartree-Fock/3-21G(d,p) approach have been presented. MIC values for *A. niger, P. chrysogenum, C. albicans* have been determined and the relationship between MIC and spacer length has been discussed.

## 1. Introduction

Quaternary ammonium salts were introduced as antimicrobial agents by Domagk over seventy years ago [1]. The first generation of quaternary ammonium compounds (QAC) were standard benzalkonium chlorides, *i.e.,* alkylbenzyldimethyl-ammonium chloride, with specific alkyl distributions, *i.e.,* C_12_, 40%; C_14_, 50% and C_16_, 10% [2]. The second generation of QAC was obtained by substitution of aromatic rings in alkylbenzyldimethylammonium chlorides by chlorine or alkyl groups to get the products like alkyldimethylethylbenzylammonium chloride with alkyl distributions of C_12_, 50%; C_14_, 30%; C_16_, 17% and C_18_, 3%. The dual quaternary ammonium salts are the third generation of QAC. These products are a mixture of equal proportion of alkyldimethylbenzylammonium chloride with alkyl distribution C_12_, 68%; C_14_, 32% and alkyldimethylethylbenzylammonium chloride with alkyl distribution C_12_, 50%; C_14_, 30%; C_16_, 17% and C_18_, 3%. The twin chain quaternary ammonium salts, like didecyldimethylammonium chloride are the fourth generation of QAC. The concept of the synergistic combination in the dual QACs has been applied to twin chain quaternary ammonium salts. The mixture of dialkyldimethylammonium chloride (dioctyl, 25%; didecyl, 25%, octyldecyl, 50%) with benzalkonium chloride (C_12_, 40%; C_14_, 50%; C_16_, 10%) is the newest blend of quaternary ammonium salts which represents the fifth generation of QACs [2]. Because of the increasing resistance of microorganisms to commonly used disinfectants, the synthesis of new types of microbiocides is a very important topic. One of the new groups with good antimicrobial activity are cyclic quaternary ammonium salts [3]. Some cyclic quaternary ammonium salts have been obtained previously by intramolecular cyclisation of amine derivatives [4,5,6,7,8,9]. Another way, *i.e.,* reaction of alkyl halides with cyclic amines can lead to chiral cyclic quaternary ammonium salts [10].

In recent years the number of applications of quaternary ammonium salts has increased considerably. They are used as biocides [11,12,13,14,15], and phase-transfer catalysts, especially in enantioselective reactions [16,17,18,19,20,21]. Pyrrolidinium salts are analogues of oxotremorine and are used as muscarinic agonist [5]. Some of quaternary ammonium salts exist as ionic liquids, which can be used as ”green solvents” [22,23,24,25,26] and electrolytes for liquid batteries [27,28].

In this work we report the synthesis, FTIR and NMR spectroscopy, DFT calculations and antimicrobial properties of polymethylene-α,ω-bis(*N,N*-dimethyl-*N*-dodecyloammonium bromides) **1**-**5**. These compounds belong to a new class of quaternary ammonium salts, the so called gemini surfactants, where two tertiary amines are connected at the nitrogen atoms by a spacer. For symmetric gemini surfactants the notation [m-s-m] is applicable, where m is the number of carbon atoms in alkyl chain and s is a number of methylene groups in a spacer. Antimicrobial activity of [12-s-12] against *Staphylococcus aureus* depends on the length of spacer, and for s = 4 MIC is four times lower than for s = 2 [29]. Gemini alkylammonium surfactants are very promising microbiocides.

## 2. Results and Discusion

### 2.1. Synthesis

**Figure 1:**
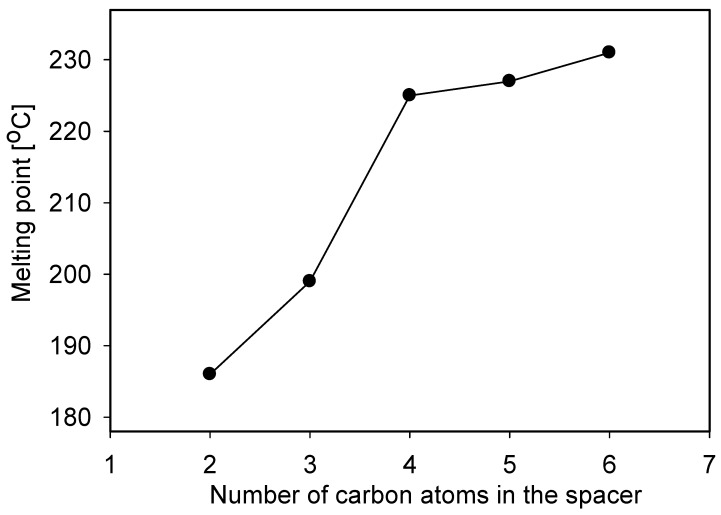
The relationship between melting points of **1-5** and number of carbon atoms in the spacer.

Polymethylene-α,ω-bis(*N,N*-dimethyl-*N*-dodecyloammonium bromides) **1-4** have been obtained by reaction of *N,N*-dimethyl-*N*-dodecylamine with 1,6-dibromohexane, 1,5-dibromopentane, 1,4-dibromo-butane and 1,3-dibromopropane, respectively. Ethylene-1,2-bis(*N,N*-dimethyl-*N*-dodecyloammonium bromide) (**5**) has been obtained by reaction of *N,N,N’,N’*-tetramethylethylenediamine with 1-bromo-decane. The reaction times were significantly shorter in comparison to procedures described in the literature, which is due to the more polar solvent that we used. The reaction yields were very high and varied from 83 to 92%. The analysis of melting points of gemini surfactants **1-5** shows the relationship between m.p. and the number of carbon atoms in the spacer (Figure 1).

A higher number of carbon atoms in the spacer corresponded to a higher melting point. This clearly suggests stronger hydrophobic interactions between the hydrocarbon chains with elongation of spacer and better packaging in the crystal. Solubility in water of polymethylene-α,ω-bis(*N,N*-dimethyl-*N*-dodecyloammonium bromides) also depends on the spacer length. Ethylene-1,2-bis(*N,N*-dimethyl-*N*-dodecyloammonium bromide) (**5**) is soluble in water below 0.1% wt./wt. while hexamethylene-1,6-bis(*N,N*-dimethyl-*N*-dodecylammonium bromide) (**1**) is readily soluble in water.

### 2.2. DFT calculations

**Figure 2:**
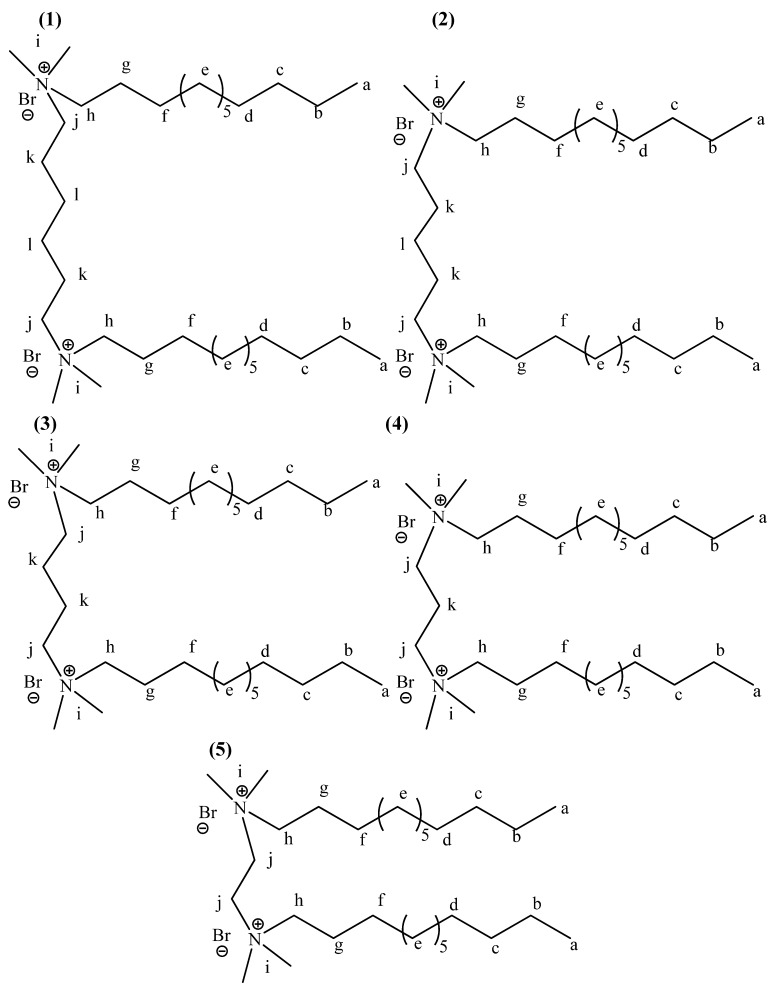
Structures and numbering of hexamethylene-1,6-bis(*N,N*-dimethyl-*N*-dodecyl-ammonium bromide) (**1**), pentamethylene-1,5-bis(*N,N*-dimethyl-*N*-dodecylammonium bromide) (**2**), tetramethylene-1,4-bis(*N,N*-dimethyl-*N*-dodecylammonium bromide) (**3**), trimethylene-1,3-bis-*N,N*-dimethyl-N-dodecylammonium bromide) (**4**) and ethylene-1,2-bis(*N,N*-dimethyl-*N*-dodecylammonium bromide) (**5**).

**Figure 3:**
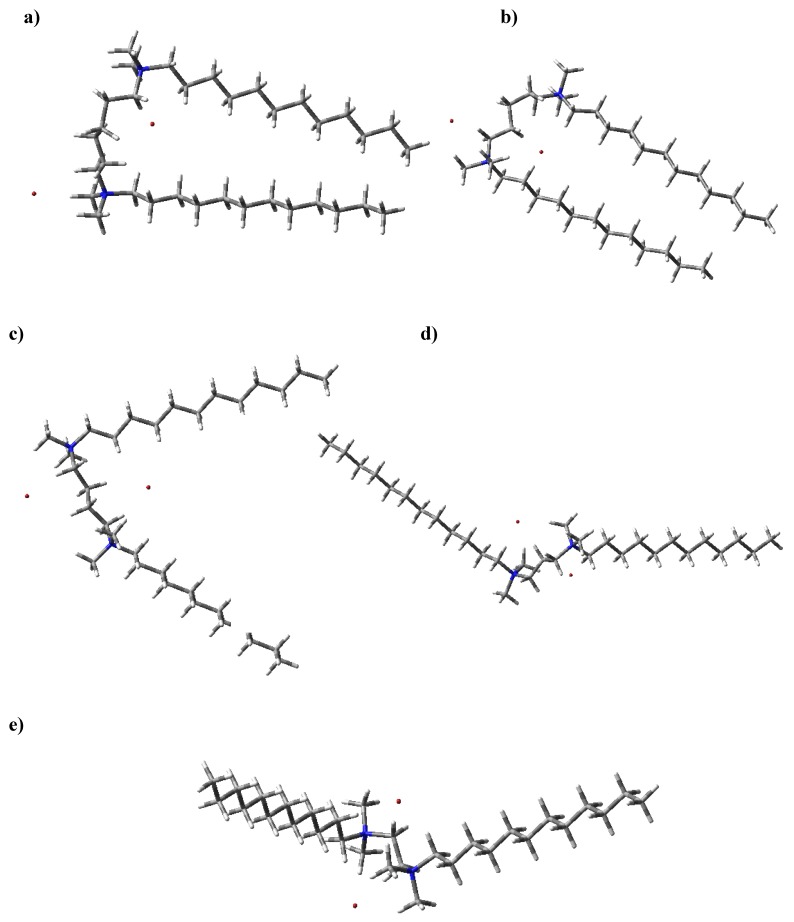
Structures of **1-5** optimized by the Hartree-Fock/3-21G(d,p) method; (a) hexamethylene-1,6-bis(*N,N*-dimethyl-*N*-dodecylammonium bromide) (**1**), (b) penta-methylene-1,5-bis(*N,N*-dimethyl-*N*-dodecylammonium bromide) (**2**), (c) tetramethylene-1,4-bis(*N,N*-dimethyl-*N*-dodecylammonium bromide) (**3**), (d) trimethylene-1,3-bis(*N,N*-dimethyl-*N*-dodecylammonium bromide) (**4**) and (e) ethylene-1,2-bis(*N,N*-dimethyl-*N*-dodecylammonium bromide) (**5**).

**Figure 4:**
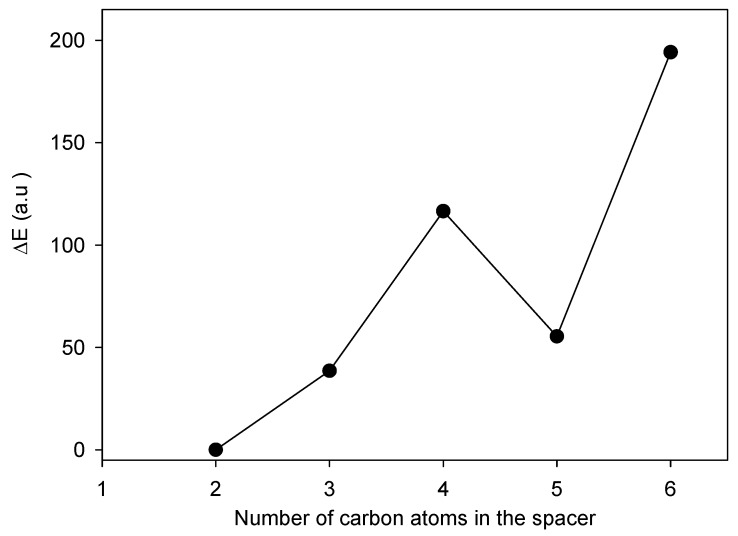
The relationship between number of carbon atoms in the spacer and relative energy ΔE (a.u.) of 1-5.

The structure and numbering for **1-5** are given in Figure 2. The structures optimized at the HartreeFock/3-21G(d,p) level of theory are shown in Figure 3. The geometry parameters, energy and dipole moments computed using the Hartree-Fock/3-21G(d,p) method are given in Table 1. The calculated energy depends on the number of carbon atoms in the spacer. The relative stabilizing energy ΔE (a.u.) is a difference between calculated energy for **5**, *i.e.,* compound with ethylene group as a spacer and for **1**, *i.e.,* a compound with a hexamethylene spacer (Table 1). According to this assumption the most stable is hexamethylene-1,6-bis(*N,N*-dimethyl-*N*-dodecylammonium bromide) (**1**, Figure 4).

The highest stabilizing energy of **1** is a consequence of intramolecular hydrophobic interactions between two alkyl chains (Figure 3a). In compound **5** with an ethylene spacer, the distance between two nitrogen atoms is 3.8 Ǻ, whereas the thickness of the dodecyl group is over 5.1 Ǻ. These distances were calculated using the data from Table 1. Because of the N···N distance and geometry conditions, the structure of **5** with an ethylene group in the spacer is much more open and two dodecane chains cannot interact each other. Thus the stabilizing energy of **5** is low. In general as the spacer becomes longer the alkyl chains get closer together and compounds are more stabilized. In case of pentamethylene-1,5-bis(*N,N*-dimethyl-*N*-dodecylammonium bromide) (**2**) the stability of the structure is slightly decreased by conformational strain (Figure 4). Additionally, bromide anions in **1-4** are engaged in three non-linear weak intramolecular interactions with carbon atoms (Figure 3, Table 1).

In **5** the bromide atom forms only two non-linear weak intramolecular C-H···Br interactions. Bromide anions interact also via Coulombic attractions with positively charged nitrogen atoms. The N^+^···Br^-^ distances are given in Table 1. The conclusions concerning stability of **1-5** in gas phase are in accordance with the stability of **1-5** in the solid state expressed by an increasing melting points for compounds with longer spacer.

### 2.3. FTIR spectra study

The FTIR spectra of polymethylene-α,ω-bis(N,N-dimethyl-N-dodecyloammonium bromides) **1-5** were measured in KBr pellets at 20 °C.

**Figure 5:**
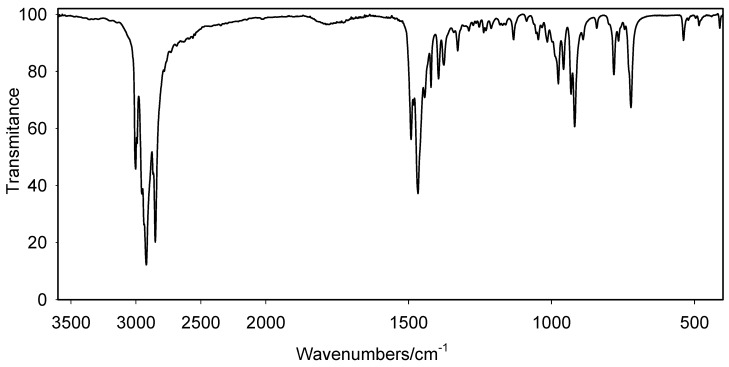
FTIR spectrum of ethylene-1,2-bis(*N,N*-dimethyl-*N*-dodecylammonium bromide) (**5**).

The spectra show typical bands of stretching asymmetric (ν_as_) and symmetric (ν_a_) vibrations as well as bands of deformation vibrations (δ), at 2850-2980 cm^-1^ and 1360-1490 cm^-1^, respectively (Figure 5). No significant changes were observed between the FTIR spectra of **1** with the longest spacer and **5** with the shortest spacer.

### 2.4. ^1^H-NMR and ^13^C-NMR spectra

**Figure 6:**
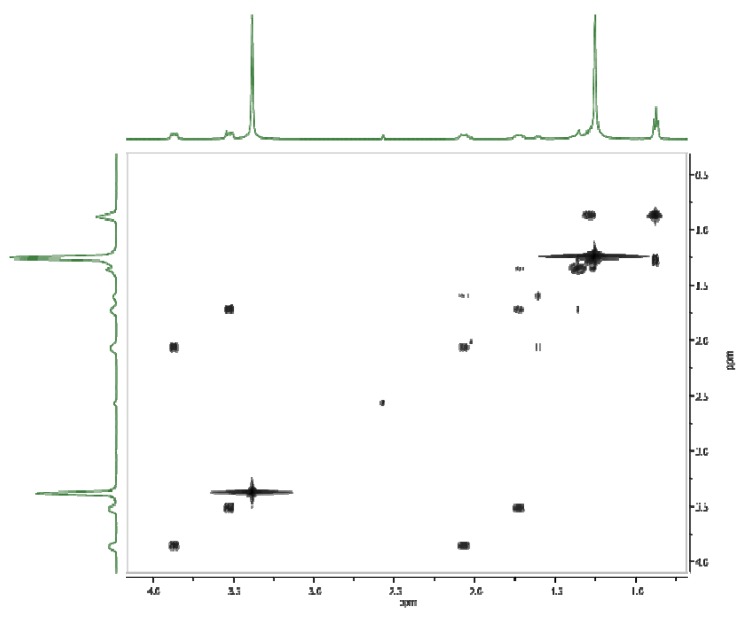
COSY NMR spectrum for hexamethylene-1,6-bis(*N,N*-dimethyl-*N*-dodecyldodecylammonium bromide) (**1**).

The proton chemical shift assignments (Table 2) of polymethylene-α,ω-bis(*N,N*-dimethyl-*N*-dodecyloammonium bromides) **1-5** are based on 2D COSY experiments, in which the proton-proton connectivity is observed through the off-diagonal peaks in the counter plot (Figure 6). The relations between the experimental ^1^H and ^13^C chemical shifts (δ_exp_) and the GIAO (Gauge-Independent Atomic Orbitals) isotropic magnetic shielding tensors (σ_calc_) are shown in Figure 5 and Figure 6. Both correlations are linear, described by the relationship: δ_exp_ = a + b·σ_calc_ The a and b parameters are given in Table 2. It has been reported in the literature [20] that the correlation between the experimental chemical shifts and calculated isotropic screening constants are usually better for carbon-13 atoms than for protons. The protons are located on the periphery of the molecule, thus are more sensitive to solute-solvent interactions than carbon atoms which are more hidden. For this reason the correlation between the experimental and calculated data for protons is worse than that for carbon atoms. The differences between calculated and experimental shifts for protons and carbons are due to different phases. The calculated shifts describe single molecules in the gas phase, while experimental shifts include all interactions in the condensed phase.

### 2.5. Antimicrobial activity

Minimal inhibitory concentration (MIC) values for all polymethylene-α,ω-bis(*N,N*-dimethyl-*N*-dodecyloammonium) bromides against *A. niger* ATCC 16404 and *A. niger* LOCK 0439 are higher than those against *P. chrysogenum* LOCK 0531 and *C. albicans* ATCC 10231 (Table 3).

**Figure 9:**
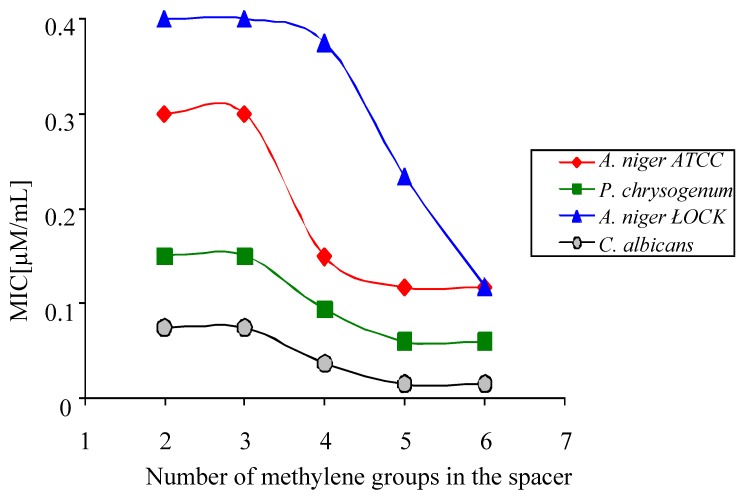
The relationship between number of methylene groups in the spacer of polymethylene-α,ω-bis(*N,N*-dimethyl-*N*-dodecyloammonium bromides) **1-5** and MIC of conidia.

*Aspergillus niger* is a fungus, which are very resistant to chemical disinfectants, what make them very difficult to removed from the surface and air. The highest antifungal activity was shown by hexamethylene-1,6-bis(*N,N*-dimethyl-*N*-dodecylammonium bromide) (**1**) and pentamethylene-1,5-bis(*N,N*-dimethyl-*N*-dodecylammonium bromide) (**2**). In general for all fungal strains a longer spacer corresponds to a lower MIC (Figure 9). This phenomenon may result from the fact that polymethylene-α,ω-bis(*N,N*-dimethyl-*N*-dodecyloammonium bromides) with long spacers are more flexible and connect more easily with the conidial surface. The MIC value of dodecyltrimethylammonium chloride, which is a monomer analog of polymethylene-α,ω-bis(*N,N*-dimethyl-*N*-dodecyloammonium bromides), against *A. niger* is 20 times higher than the MIC value for hexamethylene-1,6-bis(*N,N*-dimethyl-*N*-dodecylammonium bromide) (**1**) [2]. This means that the same biocidal effect can be obtained using at least ten times less dimeric surfactant instead of a monomeric surfactant. These are fundamental results for the application of gemini surfactants like polymethylene-α,ω-bis(*N,N*-dimethyl-*N*-dodecyloammonium bromides), as chemical microbiocides.

The minimal concentrations which inhibit 48-hour mycelium development for hexamethylene-1,6-bis(*N,N*-dimethyl-*N*-dodecylammonium bromide) (**1**) and pentamethylene-1,5-bis(*N,N*-dimethyl-*N*-dodecylammonium bromide) (**2**) are significantly higher than those for conidia. (Table 4). The higher MIC values for mycelium in comparison to conidia result from the character of mycelium growth. Fragments of hypha growing by apical elongation are sensitive to disinfectants, while portions of the hyphae away from the tips are more resistant [30]. This resistance is due to a thicker cellular wall that hinders penetration of microbiocide molecules. Another reason of this resistance is wall porosity, which decreases with age [31].

## 3. Experimental 

### 3.1. General

The NMR spectra were measured with a Varian Gemini 300VT spectrometer, operating at 300.07 and 75.4614 MHz for ^1^H and ^13^C, respectively. Typical conditions for the proton spectra were: pulse width 32°, acquisition time 5s, FT size 32 K and digital resolution 0.3 Hz per point, and for the carbon spectra pulse width 60°, FT size 60 K and digital resolution 0.6 Hz per point, the number of scans varied from 1,200 to 10,000 per spectrum. The ^13^C and ^1^H chemical shifts were measured in CDCl_3_ relative to an internal standard of TMS. All proton and carbon-13 resonances were assigned by ^1^H (COSY) and ^13^C (HETCOR). All 2D NMR spectra were recorded at 298 K on a Bruker Avance DRX 600 spectrometer operating at the frequencies 600.315 MHz (^1^H) and 150.963 MHz (^13^C), and equipped with a 5 mm triple-resonance inverse probe head [^1^H/^31^P/BB] with a self-shielded *z* gradient coil (90° ^1^H pulse width 9.0 μs and ^13^C pulse width 13.3 μs). Infrared spectra were recorded in the KBr pellets using a FT-IR Bruker IFS 66 spectrometer. The ESI (electron spray ionization) mass spectra were recorded on a Waters/Micromass (Manchester, UK) ZQ mass spectrometer equipped with a Harvard Apparatus syringe pump. The sample solutions were prepared in methanol at a concentration of approximately 10^-5^ M. The standard ESI – MS mass spectra were recorded at a 30 V cone voltage.

### 3.2. Computational details 

The calculations were performed using the Gaussian 03 program package [32] at the Hartree-Fock [33,34] levels of theory with the 3-21 basis set [33]. The NMR isotopic shielding constants were calculated using the standard GIAO (Gauge-Independent Atomic Orbital) approach [32,33,34,35] of GAUSSIAN 03 program package [36].

### 3.3. Antimicrobial study

*Fungal strains*. The antifungal activity of the gemini surfactants was evaluated against *Aspergillus brasiliensis* (previously *A. niger*) ATCC 16404, *Aspergillus niger* LOCK 0439, *Penicillium chrysogenum* LOCK 0531 and *Candida albicans* ATCC 10231.

*Antifungal activity.* Minimal inhibitory concentrations (MIC values) against conidia of moulds were measured by a tube standard 2-fold dilution method. Malt Extract Broth (Merck) was used for the antifungal tests. Moulds were preincubated on MEA slant for 5 days at 28 ºC, yeast – for 1 day at 37 ºC. Conidia suspensions of each strain were prepared by adding sterile water containing 0.1% (w/w) Tween 80 to the slant. The yeast cell suspension of *Candida albicans* was prepared by similar procedure but without Tween 80. The conidia and yeast cells were adjusted to 1-2 × 10^6^ cells/ml by counting them in Thoma chamber. One mL of conidia suspension was mixed with 1 mL of media containing the tested compounds and incubated at 28 ºC for 72 h – moulds, 37 ºC for 48 h - yeast. The MICs were defined as the lowest concentration of the compounds at which there was no visible growth. Minimal inhibitory concentrations (MIC values) against mycelium of moulds were measured by the suspension method. Conidia and yeast cells suspensions were prepared in the same way as for the conidia test. Next, 1.5 mL of inoculum was mixed with 12 mL of MEB medium and incubated for 48 h - moulds at 28 ºC, yeast- 37 ºC. After this time, 1.5 mL of tested gemini surfactants in different concentrations were added and all cultures were incubated another 48 h. The control sample was culture of mycelium without gemini surfactants. In this case, the MICs were defined as the lowest concentration of the compounds at which the development of mycelium in comparison to the control sample was inhibited. Each experiment was repeated three times and the mean values were used to compute the MICs.

### 3.4. Synthesis 

*Hexamethylene-1,6-bis(N,N-dimethyl-N-dodecyldodecylammonium bromide)* (**1**). *N,N*-Dimethyl-*N*-dodecylamine (18.2 g, 0,09 M) was mixed with 1,6-dibromohexane (10,7 g, 0.04 M) in acetonitrile (80 mL). The reaction mixture was heated under reflux for 5 h. The solvent was evaporated under reduced pressure and the residue was dried over P_4_O_10_ and then recrystallized from acetonitrile, yield 84%, m.p. 231-232 °C; Elemental analysis: found (calc) %C 60.54 (60.88); %H 11.75 (11.12); %N 4.03 (4.18); ES^+^MS *m/z* 255 (C_34_H_74_N_2_/2); ^1^H-NMR: δ 0.88 [6H, H(a)], 1,25 [4H, H(b)], 1.25 [4H, H(c)], 1.25 [4H, H(c)], 1.25 [4H, H(d)], 1.25 [20H, H(e)], 1.25 [4H, H(f)], 1.72 [4H, H(g)], 3.52 [4H, H(h)], 3.39 [12H, H(i)], 3.69 [4H, H(j)], 1.98 [4H, H(k)], 1.56 [4H, H(l)]; ^13^C-NMR: δ 13.84 C(a), 22.63 C(b), 31.60 C(c), 29.17 C(d), 29.17 C(e), 26.06 C(f), 22.38 C(g), 64.38 C(h), 50.73 C(i), 63.81 C(j), 21.51 C(k), 24.42 C(l)

*Pentamethylene-1,5-bis(N,N-dimethyl-N-dodecyldodecylammonium bromide)* (**2**). *N,N*-Dimethyl-dodecylamine (9.3 g, 0.04 M) was mixing with 1,5-dibromopentane (5.0 g, 0.02 M) in acetonitrile (80 mL). The reaction mixture was heated under reflux for 4 h. The solvent was evaporated under reduced pressure and the residue was dried over P_4_O_10_ and then recrystallized from acetonitrile, yields 86%, m.p. 226-227 °C; Elemental analysis found (calc) %C 59.99 (60.35); %H 11.58 (11.05); %N 4.02 (4.27); ES^+^MS *m/z* 248 (C_33_H_72_N_2_/2); ^1^H-NMR: δ 0.88 [6H, H(a)], 1,36 [4H, H(b)], 1.25 [4H, H(c)], 1.25 [4H, H(c)], 1.25 [4H, H(d)], 1.25 [20H, H(e)], 1.25 [4H, H(f)], 1.73 [4H, H(g)], 3.52 [4H, H(h)], 3.38 [12H, H(i)], 3.86 [4H, H(j)], 2.08 [4H, H(k)], 1.62 [2H, H(l)]; ^13^C-NMR: δ 13.98 C(a), 22.79 C(b), 31.75 C(c), 29.28 C(d), 29.28 C(e), 26.20 C(f), 22.46 C(g), 64.45 C(h), 50.69C(i), 63.76 C(j), 21.73 C(k), 22 53 C(l).

*Tetraethylene-1,4-bis(N,N-dimethyl-N-dodecyldodecylammonium bromide)* (**3**). *N,N*-Dimethyl-dodecylamine (9.7 g, 0.05 M) was mixing with 1,4-dibromobutane (4.9 g 0.03 M) in acetonitrile (80 mL). The reaction mixture was heated under reflux for 5 h. The solvent was evaporated under reduced pressure and the residue was dried over P_4_O_10_ and then recrystallized from mixture acetonitrile:aceton, yields 92%, m.p. 225-226 °C; Elemental analysis found (calc) %C 58.74 (59.80); %H 11.47 (10.98); %N 4.18 (4.36); ES^+^MS *m/z* 241(C_32_H_70_N_2_/2); ^1^H-NMR: δ 0.88 [6H, H(a)], 1,26 [4H, H(b)], 1.26 [4H, H(c)], 1.26 [4H, H(c)], 1.26 [4H, H(d)], 1.26 [20H, H(e)], 1.26 [4H, H(f)], 1.76 [4H, H(g)], 3.46 [4H, H(h)], 3.33 [12H, H(i)], 3.85 [4H, H(j)], 2.08 [4H, H(k)]; ^13^C-NMR: δ 13.81 C(a), 22.60 C(b), 31.59 C(c), 29.19 C(d), 29.19 C(e), 26.08 C(f), 22.37 C(g), 64.75 C(h), 50.67 C(i), 63.17C(j), 19.57 C(k).

*Threemethylene-1,3-bis(N,N-dimethyl-N-dodecyldodecylammonium bromide)* (**4**). *N,N*-dimethyl-dodecylamine (9.7 g, 0.05 M) was mixing with 1,3-dibromopentane (4.6 g, 0.03 M) in acetonitrile (80 mL). The reaction mixture was heated under reflux for 6 h. The solvent was evaporated under reduced pressure and the residue was dried over P_4_O_10_ and then recrystallized from mixture acetonitrile:aceton, yields 86%, m.p. 199-200 °C; Elemental analysis found (calc) %C 58.81 (59.22); %H 10.56 (10.90); %N 4.17 (4.46); ES^+^MS *m/z* 234 (C_31_H_68_N_2_/2); ^1^H-NMR: δ 0.88 [6H, H(a)], 1,35 [4H, H(b)], 1.26 [4H, H(c)], 1.26 [4H, H(d)], 1.26 [20H, H(e)], 1.26 [4H, H(f)], 1.79 [4H, H(g)], 3.55 [4H, H(h)], 3.42 [12H, H(i)], 3.82 [4H, H(j)], 2.77 [4H, H(k)]; ^13^C-NMR: δ 13.87 C(a), 22.73 C(b), 31.66 C(c), 29.27 C(d), 29.27 C(e), 26.13 C(f), 22.42 C(g), 66.00 C(h), 50.99, C(i), 60.64 C(j), 18.55 C(k).

*Ethylene-1,2--bis (N,N-dimethyl-N-dodecyldodecylammonium bromide)* **(5)**. *N,N,N’,N’*-Tetra-methylethylamine (2 g, 0.02 M) was mixing with bromododecane (4.6 g, 0.03 M) in acetonitrile (30 mL). The reaction mixture was heated under reflux for 4 h. The solvent was evaporated under reduced pressure and the residue was dried over P_4_O_10_ and then recrystallized from mixture ethanol:ethyl acetate, yields 83%, m.p. 186-187 °C; Elemental analysis found (calc) %C 58.49 (58.62); %H 10.83 (10.82); %N 4.57 (4.56); ES^+^MS *m/z* 227 (C_30_H_66_N_2_/2); ^1^H-NMR): δ 0.88 [6H, H(a)], 1,30 [4H, H(b)], 1.25 [4H, H(c)], 1.25 [4H, H(d)], 1.25 [20H, H(e)], 1.25 [4H, H(f)], 1.81 [4H, H(g)], 3.67 [4H, H(h)], 3.43 [12H, H(i)], 4.55 [4H, H(j)]; ^13^C-NMR (CDCl_3_): δ 14.03 C(a), 22.99 C(b), 31.84 C(c), 29.53 C(d), 29.53 C(e), 26.20 C(f), 22.60 C(g), 65.54 C(h), 51.29, C(i), 56.51 C(j).

## 4. Conclusions

Polymethylene-α,ω-bis(*N,N*-dimethyl-*N*-dodecyloammonium bromides) **1-4** have been obtained with good yield after short reaction times by reaction of *N,N*-dimethyl-*N*-dodecylamine with 1,6-dibromohexane, 1,5-dibromopentane, 1,4-dibromobutane and 1,3-dibromopropane. Ethylene-1,2-bis-(*N,N*-dimethyl-*N*-dodecyloammonium bromide) (**5**) has been prepared in good yield by reaction of *N,N,N’,N’*-tetramethylethylenediamine with 1-bromodecane. The structures of the title compounds have been analyzed by FTIR and NMR spectroscopy, as well as by DFT calculations. Properties of polymethylene-α,ω-bis(*N,N*-dimethyl-*N*-dodecyloammonium bromides) **1-5**, including energy, melting points, solubility and antifungal activity, depend strongly on the length of the spacer. The longer the spacer the better the water solubility, stability and antifungal activity. Hexamethylene-1,6-bis(*N,N*-dimethyl-*N*-dodecyloammonium bromide) shows the best antifungal activity and can be used as an efficient microbiocide.

## Figures and Tables

**Figure 7:**
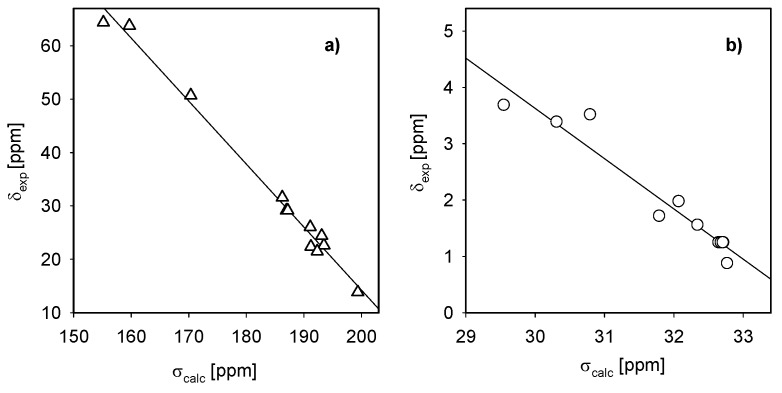
Experimental chemical shifts (δ_exp_, CD_3_Cl) in hexamethylene-1,6-bis(*N,N*-dimethyl-*N*-dodecylammonium bromide) (**1**) *vs*. the isotropic magnetic shielding (σ_calc)_ from the GIAO/HF/3-21G(d,p) calculations for molecules δ_exp_ = a + b·σ_calc_ : (a) ^13^C and (b) ^1^H.

**Figure 8:**
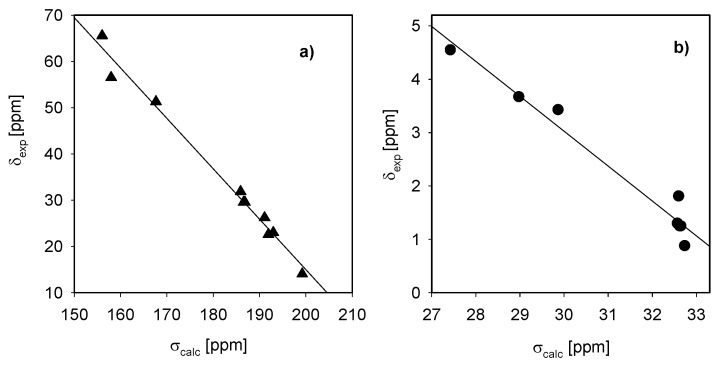
Experimental chemical shifts (δ_exp_, CD_3_Cl) in ethylene-1,2-bis(*N,N*-dimethyl-*N*-dodecylammonium bromide) (**5**) *vs.* the isotropic magnetic shielding (σ_calc)_ from the GIAO/HF/3-21G(d,p) calculations for molecules δ_exp_ = a + b σ_calc_ : (a) ^13^C and (b) ^1^H.

**Table 1:** Selected parameters of investigated compounds **1-5** calculated by the Hartree- Fock/3-21G(d,p) method.

Parameters	1	2	3	4	5
Energy (a.u)	-6551.697749	-6412.885113	-6474.064183	-6396.071637	-6357.506938
Dipole moment (Debye)	20.4558	17.1183	4.7632	7.1234	6.5095
*Bond length (Å)*					
N^+^…Br^-^	3.895 4.235	3.932 3.902	3.862 3.954	4.116 4.116	3.638 3.768
C(i)-H…Br^-^	3.681	3.727	3.674	3.297	3.383
C(j)-H…Br^-^	3.859	3.551	3.477	3.213	3.111
C(l)-H…Br^-^	3.851			1.550	
C(h)-H…Br^-^		3.661	4.003		
N-C(h)	1.537	1.536	1.534		1.550
N-C(i)	1.506	1.501	1.503	1.536	1.529
N-C(j)	1.520	1,540	1.539	1.541	1.550
C(j)-C(k)	1.540	1.538	1.528		
C(h)-C(g)	1.533	1.535	1.533	1.541	1.544
*Bond angle (^o^)*					
N-C(h)-C(g)	115.7	116.4	116.2	114.5	114.5
N-C(j)-C(k)	115.0	116.7	115.7	110.2	-
C(j)-N-C(h)	110.0	113.0	111.6	108.2	110.8
C(i)-N-C(h)	107.1	108.3	108.1	110.6	113.4
*Dihedral angle (^o^)*					
N-C(h)-C(g)-C(f)	167.4	177.8	179.9	-179.4	-174.0
N-C(j)-C(k)-C(l)	160.0	-158.9	-179.9	-	-
C(i)-N-C(h)-C(g)	168.7	56.6	175.4	-69.7	-49.1
C(i)-N-C(j)-C(k)	43.7	-176.4	148.5	161.4	-
C(a)-C(b)-C(c)-C(d)	179.7	-179.6	180.0	180.0	180.0
C(j)-N-C(h)-C(g)	47	172.1	58.6	172.9	-166.2

**Table 2:** Chemical shifts (δ, ppm) in CD_3_Cl calculating GIAO nuclear magnetic shielding tensors (σ_calc_) for hexamethylene-1,6-bis(*N,N*-dimethyl-*N*-dodecylammonium bromide) (**1**) and ethylene-1,2-bis(*N,N*-dimethyl-*N*-dodecylammonium bromide) (**5**). The predicted GIAO chemical shifts were computed from the linear equation δ_exp._ = a + b·σ_calc_ with a and b determined from the fit the experimental data ( r is the correlation coefficient).

	δ_exp._	δ_calc_	σ_calc_		δ_exp._	δ_calc_	σ_calc_
Hexamethylene-1,6-bis(N,N-dimetyl-N-dodecyldodecylammonium bromide) (**1**)							
*Carbon-13*				*Proton*			
C (a)	13.84	14.98	199.37	H (a)	0.88	1.16	32.767
C (b)	22.63	21.93	193.49	H (b)	1.25	1.27	32.644
C (c)	31.60	30.49	186.25	H(c)	1.25	1.21	32.711
C (d)	29.17	29.62	186.98	H (d)	1.25	1.22	32.699
C (e)	29.17	29.35	191.11	H (e)	1.25	1.24	32.676
C (f)	26.06	24.74	191.23	H (f)	1.25	1.22	32.704
C(g) C(h) C(i) C(j) C(k) C(l) a	22.38 64.38 50.73 63.81 21.51 24.42	24.60 67.19 49.27 61.86 23.33 22.38 250.5599	155.19 170.35 159.70 192.31 193.11	H(g) H(h) H(i) H(j) H(k) H(l)	1.72 3.52 3.39 3.69 1.98 1.56	2.03 2.92 3.35 4.03 1.78 1.54 30.40303	31.787 30.790 30.308 29.54 732.06 832.339
b		-1.1816				-0.892456	
r^2^		0.98999				0.93883	
Ethylene-1,2-bis-(N,N-dimethyl-N-dodecylammonium bromide) (**5**)							
*Carbon-13*				*Proton*			
C (a)	14.3	15.84	199.21	H (a)	0.88	1.24	32.732
C (b)	22.99	22.64	192.97	H (b)	1.30	1.35	32.568
C (c)	31.84	30.32	185.92	H(c)	1.25	1.29	32.648
C (d)	29.53	29.62	186.56	H (d)	1.25	1.30	32.637
C (e)	29.53	29.31	186.85	H (e)	1.25	1.31	32.620
C (f)	26.20	24.67	191.10	H (f)	1.25	1.31	32.623
C(g) C(h) C(i) C(j) a	22.60 65.54 51.29 56.51	23.77 62.89 50.24 60.78 232.9141	191.93 156.04 167.64 157.57	H(g) H(h) H(i) H(j)	1.81 3.67 3.43 4.55	1.33 3.69 3.11 4.71 22.6482	32.598 28.976 29.866 27.423
b		-1.0897				-0.6541	
r^2^		0.98592				0.96714	

**Table 3:** The minimal inhibitory concentration (MIC) (μM/mL) of polymethylene-α,ω-bis(N,N-dimethyl-N-dodecyloammonium bromides) (**1-5**) for conidia and vegetative cells.

Compound	Strains			
	*A.niger* ATCC 16404	*P.chrysogenum* LOCK 0531	*A.niger* LOCK 0439	*C.albicans* ATCC 10231
(**1**)	0.12	0.06	0.12	0.015
(**2**)	0.12	0.06	0.24	0.015
(**3**)	0.15	0.095	0.375	0.037
(**4**)	0.3	0.15	0.4	0.075
(**5**)	0.3	0.15	0.4	0.075

**Table 4:** (MIC, μM/mL) of hexamethylene-1,6-bis(*N,N*-dimethyl-*N*-dodecylammonium bromide) (**1**) and pentamethylene-1,5-bis(*N,N*-dimethyl-*N*-dodecylammonium bromide) (**2**) for mycelium and pseudomycelium.

Compound	Strains			
	*A.niger* ATCC 16404	*P.chrysogenum* LOCK 0531	*A.niger* LOCK 0439	*C.albicans* ATCC 10231
**1**	0.31	0.31	0.31	0.31
**2**	0.31	0.76	0.76	0.45
